# Supplementary figures and images for: ReFeaFi: Genome-wide prediction of regulatory elements driving transcription initiation
Source: PLoS Comput Biol. 2021 Sep 7;17(9):e1009376. doi: 10.1371/journal.pcbi.1009376 (PMC8448322; doi:10.1371/journal.pcbi.1009376)

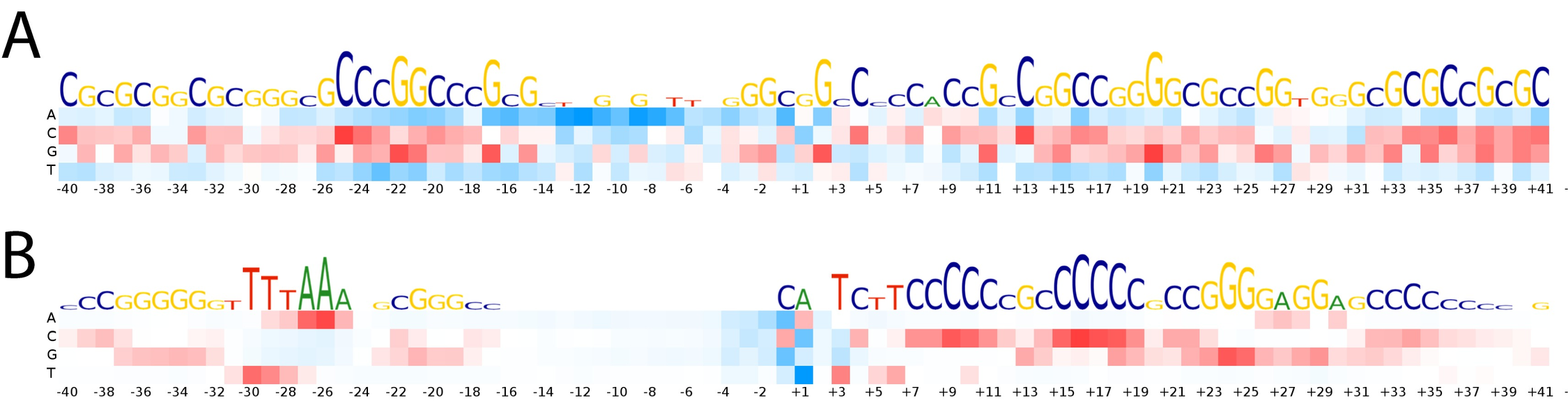

Supplement: S1 Fig — a. Random negatives model. b. Difficult negatives model. (TIF) [file pcbi.1009376.s001.tif]

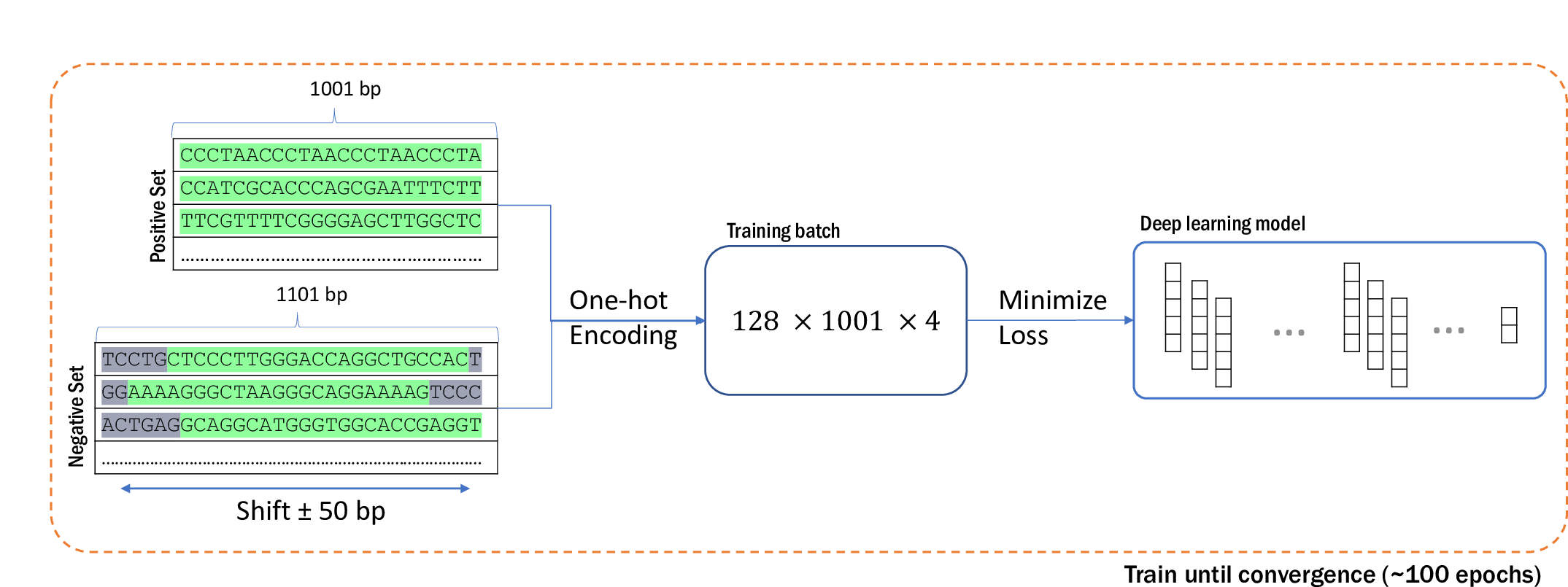

Supplement: S2 Fig — (TIF) [file pcbi.1009376.s002.tif]

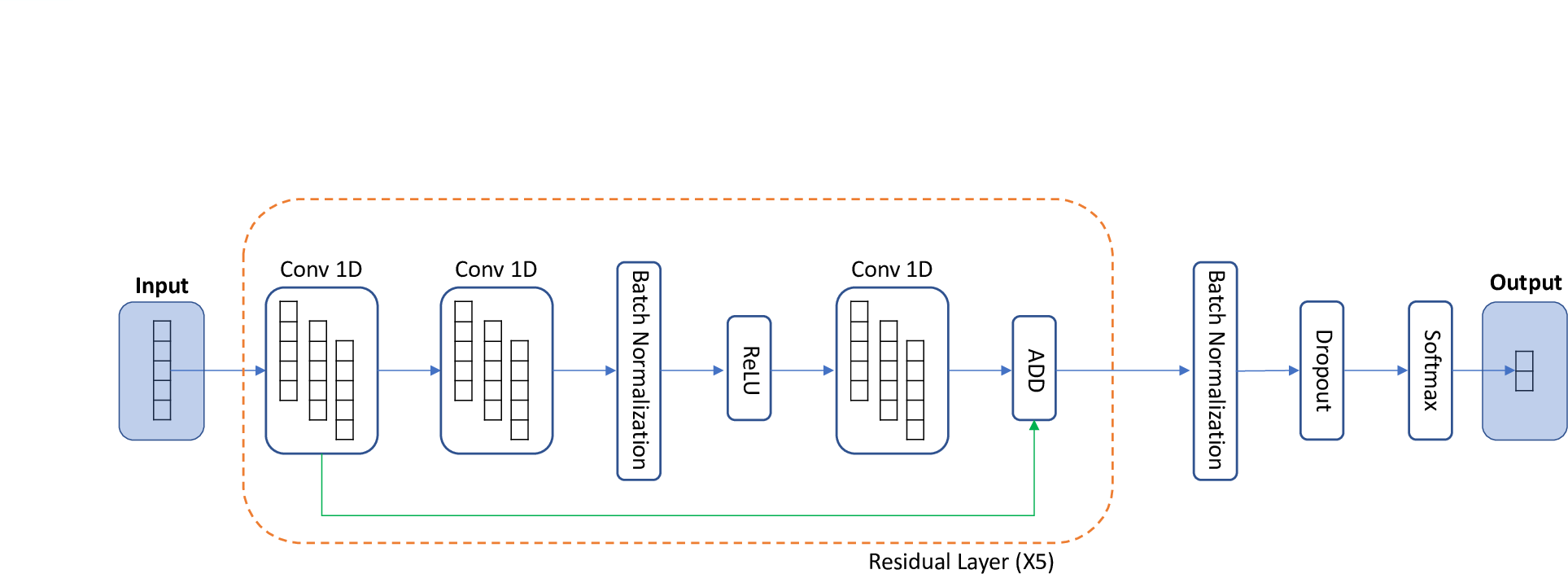

Supplement: S3 Fig — The model employs CNN with residual connections which is followed by a softmax layer. (TIF) [file pcbi.1009376.s003.tif]
